# The association between contact with children and the clinical course of COVID-19

**DOI:** 10.1017/S0950268822000474

**Published:** 2022-03-07

**Authors:** Peter Jannuzzi, Gregory A. Panza

**Affiliations:** 1 Integrated Care Partners, Hartford HealthCare, Hartford, CT, USA; 2 Unionville Pediatrics, LLC, Unionville, CT, USA; 3 Department of Research, Hartford HealthCare, Hartford, CT, USA

**Keywords:** Coronavirus 2019, hospitalization, pandemic, SARS-CoV-2

## Abstract

We examined the association between contact with children and the clinical course of COVID-19 among COVID-19-positive adult patients. Participants completed a survey to assess demographics, medical information related to their COVID-19 diagnosis, contact with children at home and at the workplace. Patients were aged 45.68 ± 14.38 years, mostly female (72.1%), 842 were not hospitalized and 167 were hospitalized. At home, there were no differences between groups for the number of child contact hours or total child hours (hours × number of children) per week (*P*s > 0.05). The number of children at home was greater among patients not hospitalized (*P* < 0.05), however this was no longer significant after controlling for covariates (*P* > 0.05). At the workplace, there were no differences between groups (all *P*s > 0.05). Sub-group analysis found the proportion of patients that were treated in the intensive care unit (ICU) was greater among patients with no child contact (*P* < 0.05). A secondary analysis found that patients with no child contact had an increased likelihood of thromboembolism (*P* < 0.05) and a trend towards more overall COVID-19-related complications (*P* = 0.076). Overall, an association between contact with children and hospitalization was not found when adjusting for covariates. Sub-group analysis indicated a possible protective effect for more severe disease; however, these findings need further study.

## Introduction

Severe illness due to SARS-CoV-2, the virus that causes coronavirus 2019 (COVID-19) is less frequent in children compared to adults [1–3]. There are several hypotheses that may explain the nature of this resilience in children. Examples include (1) less affinity for the angiotensin-converting enzyme-2 (ACE-2) receptor sites [3]; (2) better immune modulation relating to strong T-cell response [4]; (3) younger, healthier lungs; and (4) prior minor coronavirus infection leading to immunity or resistance to the novel virus [3, 5–7]. Grifoni *et al*. [8] found that up to 60% of adults that had never been exposed to SARS-CoV-2 had an innate T-cell ability to fight the novel virus. Ng *et al*. [9] detected SARS-CoV-2 reactive antibodies in blood donors from 2015 to 2018 and were particularly prevalent in children and adolescents. This finding was further confirmed by Ladner *et al*. [10]. Bonifacius *et al*. [11] confirmed that T-cell responses to endemic viruses were robust and cross-reactive to SARS-CoV-2 epitopes despite declining humoral responses. Moreover, Wang *et al*. [12] showed that T-cell memory can be elicited by exposure to SARS-CoV-2 even in the absence of infection indicating the same response might be seen with more minor coronavirus infections among adults in contact with children harboring those viruses, even in the absence of clinical disease. The results from these studies may support the hypothesis that prior coronavirus infections lead to pre-existing immunity or resistance to SARS-CoV-2.

It is well established that children get considerably more upper respiratory infections than adults. Parents and those who work closely with children, particularly children under 3 years old, are likely to be exposed to the same illnesses as the children they care for *vs.* those that do not [13]. Therefore, if the diminished illness severity due to COVID-19 in children is related to pre-existing immunity, the severity of COVID-19 symptoms in adults may be associated with their contact with children. Yang *et al*. [14] recently found robust cross-reactive B-cell response from prior coronavirus exposures in pre-pandemic pediatric blood samples. They concluded that those responses may be responsible for the varying observed susceptibilities observed in pediatric patients with COVID-19. Yang *et al*. further noted that the responses were distinctly more prominent in children *vs.* the adults in the study; however, they did not measure how much contact adults had with children.

The association between contact with children and the severity of COVID-19 has recently been examined. Dugas *et al*. [15] found an association between milder disease and contact with children. However, the study had several limitations including very few severe cases surveyed, limited data on the degree of contact with children, lack of controlling for covariates including underlying conditions, non-representation of the general population and reliance solely on self-reporting *vs.* chart review. Furthermore, the authors based their conclusion of an association of milder disease and contact with children on only two descriptive analysis results. That is, 6.9% of their cohort had a predominately mild course of COVID-19, and their cohort reported frequent and regular job-related contact to children below 10 years of age [16]. Wood *et al*. [17] found an association between contact with children and decreased risk of any COVID-19 infection as well as a trend toward decreased rates of hospitalization. However, this study relied solely on self-report, used population-based data rather than study-level data for covariate analysis, imputed ethnicity via surname and did not include important potential covariates and confounders (e.g. body mass index (BMI), exercise habits, hypertension) of the clinical course of COVID-19. In addition, the severity of hospitalized patients was not assessed (e.g. admitted to the intensive care unit (ICU)).

The purpose of this study was to examine the association between contact with children and the clinical course of COVID-19 among COVID-19-positive patients who were hospitalized *vs.* patients who were not hospitalized. We hypothesised that patients with COVID-19 that were not hospitalized would have greater contact with children (e.g. regular household or occupational contact) compared to patients with COVID-19 who were hospitalized. The current study sought to eliminate or improve upon limitations of previous studies on this topic by implementing objective verification of survey data and controlling for potential covariates and confounders that may influence the clinical course of COVID-19.

## Methods

### Recruitment of participants

Patients were eligible for enrolment if they were aged ⩾18 years, were tested at any of the Hartford HealthCare testing sites across the state of Connecticut and subsequently diagnosed with COVID-19 between 1 January 2020 and 31 April 2021. Testing was available to any Connecticut resident, and participants were recruited via the distribution of a study flyer to patients in MyChart who tested positive for COVID-19. The flyer was re-sent twice (2 months apart) to any patients who did not view the consent form after the initial distribution of the flyer. The flyer included a brief description of the study including time commitment, the study investigator's names, purpose of the study, as well as a link to REDCap. The REDCap link included the study consent, HIPAA form and the open study survey. All patients enrolled had their COVID-19 testing done by the hospital system, which enabled additional confirmation of COVID-19 diagnosis in the patient's electronic medical record. Protected health information was used to cross-reference information collected on the survey. Only investigators named on the study had access to the data and data were collected and stored in password-protected databases on a secure network drive. The Hartford HealthCare Institutional Review Board reviewed and approved the study prior to study recruitment.

### Study survey

Once enrolled, participants were asked to complete an electronic survey in REDCap (full survey in Supplementary material). The survey consisted of 25 questions, including questions for demographics (e.g. age, gender), medical information related to their COVID-19 diagnosis (e.g. symptoms, hospitalization, complications, treatment), contact with children (e.g. hours per week, number of children) at the workplace and contact with children at home. REDCap has the ability to allow users to review and change answers on the survey before submitting. Answers on the survey were cross-referenced for any variables that were also included in the patient's charts. Variables that were confirmed using the patient's chart for both inpatient and outpatients included age, gender, date of COVID-19 diagnosis (positive test), symptoms and complications related to COVID-19, and medical conditions at the time of diagnosis. Variables confirmed for inpatients included hospitalization, number of days hospitalized and treatment while being hospitalized. For any discrepancies or missing data observed, patients were contacted by the study coordinator to clarify the correct information. Vaccination status prior to diagnosis was also obtained from the patient's chart. All patients who enrolled and completed the survey were entered into a random drawing for a chance to win one of 20 iPads.

### Statistical analysis

Data were checked for normality using histograms, normal probability plots and Kolmogorov test statistics. Patient characteristics were compared between hospitalized and non-hospitalized patients using independent samples *t*-test for continuous variables. The *χ*^2^ test or Fisher's exact test was used for categorical variables. The primary outcomes of interest were contact with children at home as well as contact with children at the workplace. For these primary outcomes, hours per week, number of children and total child hours per week (hours × number of children) were compared between hospitalized and non-hospitalized patients using independent samples *t*-test, as well as multivariable logistic regression to control for relevant covariates including age, gender, BMI, exercise behavior prior to diagnosis and underlying health conditions. Contact type (i.e. home only, workplace only, both, or no contact) was also compared using a *χ*^2^ analysis as well as multivariable logistic regression to adjust for covariates. Primary outcomes were re-analyzed after removing patients who were vaccinated to examine if vaccine status influenced the results.

A secondary analysis was conducted to compare COVID-19-related complications (e.g. pneumonia, respiratory failure) between patients who had no contact and those who had any contact with children. Finally, a sub-group analysis was conducted among the hospitalized patients to further examine case severity, and examined if hospital length of stay as well as treatment differed between patients who had no contact *vs.* patients who had any contact with children. Length of stay was not normally distributed and therefore a Mann–Whitney *U* test was used to compare this variable between groups. SPSS Version 26.0 was used for all analyses, and *P* < 0.05 was established as the level of statistical significance.

## Results

### Participants

**Fig. 1.**
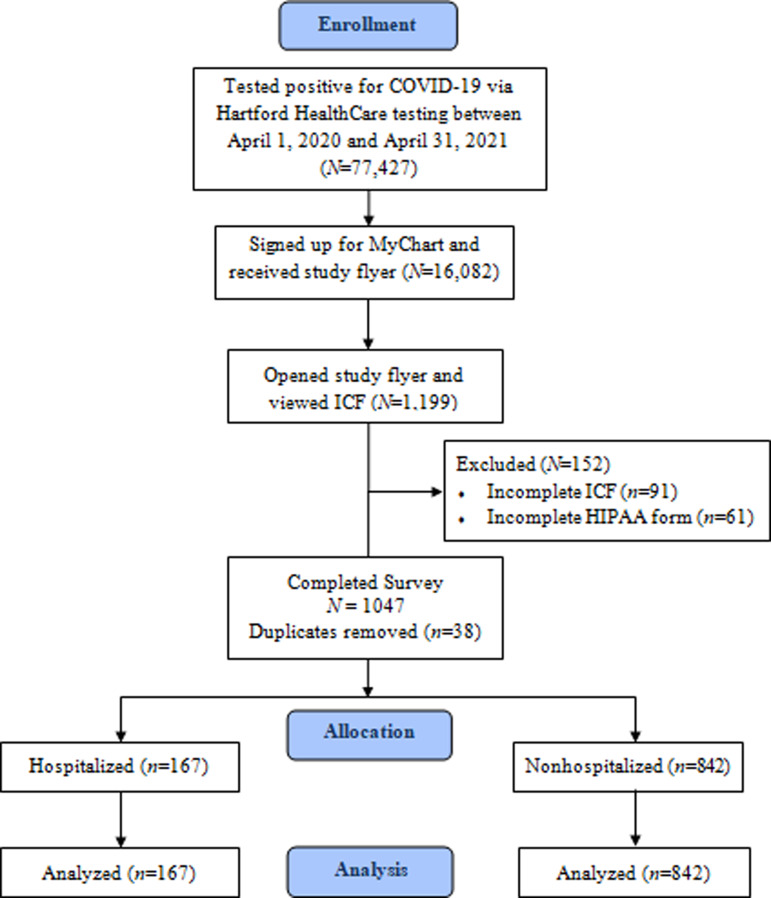
Study flow diagram.

A study enrolment flow diagram is displayed in Figure 1. The current study aimed to recruit all patients with a MyChart account who tested positive for COVID-19 via Hartford HealthCare testing between 1 April 2020 and 31 April 2021. Hospitalized patients were admitted to any one of Hartford HealthCare's seven acute care hospitals. Including only patients who tested within the study site's healthcare system allowed the ability to use the patient's chart to objectively verify diagnosis as well as other key variables. The MyChart study advertisement was opened and the consent was viewed by 1199 patients. After excluding survey records for incomplete consent, incomplete HIPAA and removal of duplicates, 1009 (84.2%) patients were included in the analysis. Duplicates were identified by patient name on the consent, and the first record of any patient with duplicate records was used in the analysis. Patient characteristics are displayed in Table 1. On average, patients were middle aged, obese (average BMI ⩾30 kg/m^2^) and the majority were female. Patients who were hospitalised (*n* = 167) were older, had greater BMI, had greater prevalence of medical conditions at the time of diagnosis, exercised less and consisted of more males compared to patients who were not hospitalised (*n* = 842; all *P*s < 0.05).

**Table 1.** Participant characteristics

Variable	Non-hospitalized (*n* = 842)	Hospitalized (*n* = 167)	Total (*N* = 1009)	95% CI or *χ*^2^	*P*
Age, mean ± s.d.	44.43 ± 14.18	51.98 ± 13.75	45.68 ± 14.38	−9.89 to −5.20	<0.001
Gender, frequency (%)				12.86	<0.001
Female	626 (74.3)	101 (60.6)	727 (72.1)		
Male	216 (25.7)	66 (39.4)	282 (27.9)		
Height (cm), mean ± s.d.	171.87 ± 88.05	169.81 ± 10.91	173.05 ± 93.90	−11.34 to 15.46	0.763
Weight (kg), mean ± s.d.	86.76 ± 22.78	98.83 ± 28.77	88.77 ± 24.28	−16.04 to −8.10	<0.001
BMI (kg/m^2^), mean ± s.d.	30.62 ± 7.62	34.01 ± 7.85	31.18 ± 7.85	−4.66 to −2.12	<0.001
Vaccinated prior to diagnosis, frequency (%)	10 (1.2)	1 (0.6)	11 (1.1)	–	1.00
Medical conditions at time of Dx, frequency (%)					
High blood pressure	203 (24.1)	65 (38.9)	268 (26.6)	15.68	<0.001
Diabetes	59 (7.0)	41 (24.6)	100 (9.9)	48.04	<0.001
Asthma	163 (19.4)	45 (26.9)	208 (20.6)	4.90	0.027
Congestive heart disease	12 (1.4)	3 (1.8)	15 (1.5)	–	0.725
Autoimmune disorder	71 (8.4)	16 (9.6)	87 (8.6)	0.23	0.629
Chronic lung disease	21 (2.5)	10 (6.0)	31 (3.1)	5.71	0.017
Chronic renal failure	1 (0.1)	1 (0.6)	2 (0.2)	–	0.203
COVID-19 symptoms, frequency (%)					
Fever or chills	518 (61.5)	126 (75.4)	644 (63.8)	11.71	<0.001
Cough	525 (62.4)	125 (74.9)	650 (64.4)	9.50	0.002
Shortness of breath or difficulty breathing	395 (46.9)	139 (83.2)	534 (52.9)	73.79	<0.001
Fatigue	681 (80.9)	150 (89.8)	831 (82.4)	7.67	0.006
Muscle or body aches	577 (68.5)	123 (73.7)	700 (69.4)	1.72	0.189
Headache	611 (72.6)	105 (62.9)	716 (71.0)	6.35	0.012
New loss of taste or smell	538 (63.9)	81 (48.5)	619 (61.3)	13.93	<0.001
Sore throat	299 (35.5)	57 (34.1)	356 (35.3)	0.12	0.733
Congestion or runny nose	495 (58.8)	68 (40.7)	563 (55.8)	18.45	<0.001
Nausea or vomiting	228 (27.1)	66 (39.5)	294 (29.1)	10.45	0.001
Diarrhoea	312 (37.1)	72 (43.1)	384 (38.1)	2.17	0.141
None	23 (2.7)	0 (0.0)	23 (2.3)	–	0.031
Exercise behaviour, frequency (%)					
Regular exercise prior to COVID-19 Dx	425 (50.5)	64 (38.3)	489 (48.5)	10.90	0.004
Regular exercise following COVID-19 Dx	255 (30.3)	42 (25.1)	297 (29.4)	9.79	0.007

Fisher's exact test used when cell counts were <5.

### Primary outcomes

Results for the primary outcomes are displayed in Table 2. The most common type of contact with children was contact in the home only, followed by contact at both home and work, and contact at work only. Approximately one-third of the sample did not have any contact with children. There were no differences in contact type between groups (all *P*s > 0.05). At home, there were no significant differences between groups for the number of child contact hours per week or total child hours per week (*P*s > 0.05). There was a significant difference between groups for the number of children at home (*P* < 0.05); however, this was no longer significant after controlling for relevant covariates (*P* > 0.05). At the workplace, there were no significant differences between groups for the number of child contact hours per week, number of children or total child hours per week (*P*s > 0.05). These results did not change after removing the 12 patients that were vaccinated prior to diagnosis and re-running the primary analyses.

**Table 2.** Contact with children and the clinical course of COVID-19: non-hospitalised *vs.* hospitalised patients

Variable	Non-hospitalized (*n* = 842)	Hospitalized (*n* = 167)	Total (*N* = 1009)	*P*	*χ*^2^ or 95% CI	*P*^a^	OR^a^	95% CI for OR^a^
Contact type, frequency (%)								
Home only	346 (41.1)	63 (37.7)	409 (40.5)	0.418	0.66	0.457	0.869	0.600–1.258
Workplace only	71 (8.4)	12 (7.2)	83 (8.2)	0.592	0.29	0.913	0.963	0.492–1.885
Both occupation and home	126 (15.0)	20 (12.0)	146 (14.5)	0.316	1.01	0.485	0.823	0.478–1.420
No contact	299 (35.5)	72 (43.1)	371 (36.8)	0.063	3.47	0.196	1.277	0.882–1.849
At home contact (mean ± s.d.)								
Hours per week	29.09 ± 38.20	23.63 ± 35.52	28.19 ± 37.81	0.076	−0.58 to 11.49	0.592	0.999	0.993–1.004
Number of children	0.89 ± 1.15	0.68 ± 1.00	0.85 ± 1.13	0.021	0.03–0.38	0.300	0.912	0.767–1.085
Total child hours per week (hours × number of children)	54.21 ± 91.09	41.22 ± 77.86	52.07 ± 89.14	0.058	−0.47 to 26.44	0.560	0.999	0.997–1.002
Workplace contact (mean ± s.d.)								
Hours per week	4.19 ± 10.47	3.56 ± 9.65	4.08 ± 10.34	0.448	−1.00 to 2.26	0.795	0.998	0.979–1.016
Number of children	5.01 ± 25.31	3.01 ± 11.47	4.68 ± 23.60	0.108	−0.44 to 4.45	0.439	0.996	0.985–1.007
Total child hours per week (hours × number of children)	124.16 ± 591.52	80.32 ± 351.68	116.91 ± 559.07	0.198	−23.01 to 110.70	0.561	1.00	1.00–1.00

OR, odds ratio.

Equal variances not assumed for continuous variables.

a *P*, OR and 95% CI for OR when adjusting for age, gender, BMI, exercise behaviour prior to diagnosis, and underlying health conditions using a multivariable logistic regression. All binary (yes/no) independent variables in logistic regression coded as: 0 = no, 1 = yes; dependent variable: 0 = non-hospitalised, 1 = hospitalised.

### COVID-19-related complications

Table 3 displays COVID-19-related complications for patients who had no contact with children (*n* = 371), any contact with children (*n* = 638) and the total sample. Patients who had no contact with children were older, had a lower BMI and exercised more often than patients who had contact with children (*P*s < 0.05). There was a significantly greater proportion of patients with no children contact that had thromboembolism compared to patients who had any contact with children (*P* < 0.05). There were no significant differences for any other COVID-19-related complication (all *P*s > 0.05). However, there was a trend indicating that the proportion of patients who had any complication was greater in those who had no contact with children compared to patients who did have contact with children (*P* < 0.1).

**Table 3.** Contact with children and COVID-19-related complications

Variable	No contact (*n* = 371)	Contact (*n* = 638)	Total (*N* = 1009)	*χ*^2^ or 95% CI	*P*
*Patient characteristics*					
Age, mean ± s.d.	47.7 ± 16.74	44.76 ± 12.74	45.68 ± 14.38	0.537–4.48	0.013
Gender, frequency (%)				1.42	0.233
Female	259 (69.8)	468 (73.3)	727 (72.1)		
Male	112 (30.2)	170 (26.7)	282 (27.9)		
Height (cm), mean ± s.d.	177.68 ± 132.07	167.97 ± 10.84	171.53 ± 80.55	−3.82 to 23.23	0.159
Weight (kg), mean ± s.d.	87.57 ± 25.69	89.46 ± 23.42	88.76 ± 24.28	−5.08 to 1.31	0.246
BMI (kg/m^2^), mean ± s.d.	30.47 ± 8.06	31.59 ± 7.55	31.18 ± 7.76	−2.13 to 0.11	0.030
Medical conditions at time of Dx, frequency (%)					
High blood pressure	109 (29.4)	159 (24.9)	268 (26.6)	2.44	0.119
Diabetes	43 (11.6)	57 (8.9)	100 (9.9)	1.88	0.171
Asthma	67 (18.1)	141 (22.1)	208 (20.6)	2.30	0.129
Congestive heart disease	6 (1.6)	9 (1.4)	15 (1.5)	0.07	0.791
Autoimmune disorder	27 (7.3)	60 (9.4)	87 (8.6)	1.33	0.249
Chronic lung disease	11 (3.0)	20 (3.1)	31 (3.1)	0.02	0.884
Chronic renal failure	1 (0.3)	1 (0.2)	2 (0.2)	–	1.00
Exercise behavior, frequency (%)					
Regular exercise prior to COVID-19 Dx	200 (53.9)	291 (45.6)	491 (48.7)	6.49	0.011
Regular exercise following COVID-19 Dx	117 (31.6)	182 (28.5)	299 (29.6)	1.07	0.300
*Complication, frequency (%)*					
Pneumonia	41 (11.1)	66 (10.3)	107 (10.6)	0.13	0.719
Respiratory failure	15 (4.0)	20 (3.1)	35 (3.5)	0.59	0.444
Thromboembolism	7 (1.9)	2 (0.3)	9 (0.9)	–	0.010
Sepsis and septic shock	4 (1.1)	5 (0.8)	9 (0.9)	–	0.630
Cardiomyopathy and arrhythmia	7 (1.9)	7 (1.1)	14 (1.4)	1.08	0.300
Kidney injury	4 (1.1)	5 (0.8)	9 (0.9)	–	0.630
Gastrointestinal bleeding	0 (0.0)	2 (0.3)	2 (0.2)	–	0.281
Other	12 (3.2)	15 (2.5)	28 (2.8)	0.47	0.495
Any complication	62 (16.7)	81 (12.7)	143 (14.2)	3.12	0.076

Fisher's exact test used for any variables with a cell count less than 5.

### Hospitalised patients

Table 4 displays treatment outcomes for patients admitted to the hospital that had no contact with children (*n* = 72) *vs.* any contact with children (*n* = 95). There was no difference in hospital length of stay (days) between patients who had no contact with children and those who had contact with children. In addition, treatment was not different between the two groups, with the exception of the proportion of patients treated in the ICU. The proportion of patients that were treated in the ICU was significantly greater in patients that did not have contact with children compared to patients that did have contact with children (*P* < 0.05). This finding remained significant after adjusting for relevant covariates.

**Table 4.** Treatment in the hospital: no contact *vs.* any contact with children

Variable	No contact (*n* = 72)	Contact (*n* = 95)	Total (*N* = 167)	*U* or *χ*^2^	*P*	*P*^a^	OR^a^	95% CI for OR^a^
Length of stay (days), median, IQR	6.00, 6.00	5.00, 6.00	6.00, 6.00	2917.00	0.127	0.677	1.008	0.97–1.04
Treatment, frequency (%)								
Medication only	37 (51.4)	58 (61.1)	95 (56.9)	1.56	0.212	0.196	1.568	0.79–3.10
Medication and ventilator	9 (12.5)	8 (8.4)	17 (10.2)	0.75	0.388	0.084	0.367	0.12–1.15
Intensive care unit	21 (29.2)	15 (15.8)	36 (21.6)	4.33	0.037	0.006	0.311	0.14–0.72
Floor unit	33 (45.8)	45 (47.4)	78 (46.7)	0.04	0.844	0.389	1.351	0.68–2.68
Other	10 (13.9)	17 (17.9)	27 (16.2)	0.49	0.486	0.248	1.740	0.68–4.46

OR, odds ratio; IQR, interquartile range.

a *P*, OR, and 95% CI for OR when adjusting for age, gender, body mass index, exercise behaviour prior to diagnosis, and underlying health conditions using a multivariable logistic regression. All binary (yes/no) independent variables in logistic regression coded as: 0 = no, 1 = yes; dependent variable: 0 = no contact, 1 = contact.

## Discussion

The current study did not find an association between contact with children and rates of hospitalization when adjusting for covariates. However, our sub-group analysis indicated that the proportion of patients that were treated in the ICU was greater in patients that did not have contact with children compared to patients that did have contact with children. In addition, a secondary analysis of COVID-19-related complications found that patients with no children contact were more likely to have a thromboembolism and a trend towards having a lower rate of overall COVID-19-related complications. Contrary to our primary findings, previous studies [15, 17, 18] have found an association between contact with children and the clinical course of COVID-19. However, the current study improved upon limitations of these previous studies by collecting more extensive demographic and health data, objectively verifying these data with patient charts, and controlling for potential confounders that may influence the clinical course of COVID-19 such as age, gender, BMI, exercise habits and underlying health conditions. In such non-randomized designs, controlling for potential confounders is essential for avoiding type I error. Our results reflect this by displaying significance or trends that support a protective effect for the ‘home contact’ primary outcome, which was lost after adjusting for covariates. Therefore, it is possible that conclusions from previous studies that failed to control for relevant covariates were misleading.

Despite not finding significance for our primary outcomes, a sub-group analysis showed that contact with children was inversely related to ICU admission, indicating a possible protective effect for more severe disease. Interestingly, a trend was also observed indicating a lower proportion of COVID-19-related complications in patients with child contact. It is difficult to explain the fewer ICU admissions or a potential decreased rate of complications in patients with child contact. The theory that underlying immunity or resistance conferred from contact with children (e.g. due to children having minor coronavirus infection pre-pandemic) is supported by these observations and perhaps this protection surfaces when examining severity level of hospitalized patients. One way to further evaluate this theory would be to test hospitalised patients for T-cell responses to minor coronaviruses. Of note, the sub-group analysis sample was relatively small and may not have been adequately powered. Therefore, findings from sub-group analyses warrant further exploration in a larger sample.

One explanation for our finding that patients with child contact were less likely to have thromboembolism is the increased activity level needed to care for children decreased their risk of thromboembolism. However, interestingly, a greater proportion of patients with no children self-reported that they exercised regularly prior to diagnosis compared to patients with child contact (53.9% *vs.* 45.6%, respectively; *P* < 0.05, Table 3). Nevertheless, the number of thromboembolism cases was very small (*n* = 9) and therefore this secondary finding needs further research. Our finding that regular exercise was associated with less severe COVID-19 outcomes supports a recent study by Sallis *et al*. [19] in over 48 000 adult patients that found physical inactivity is associated with a higher risk of severe COVID-19 outcomes.

The results from this study can be used to inform ongoing policy decisions regarding adult to child interactions. Some parents [20, 21], teachers [22] and childcare workers have been fearful of children being vectors of disease despite studies suggesting they are not major drivers of the epidemic [7, 23]. Creating policies that support traditional contact appears to pose no increased risk of COVID-19 severity to adults and allows for a more normalized experience for children and those who care for them. Results from Wood *et al*. [17] and sub-analysis results from our study suggest that contact with children may be protective against disease acquisition and more severe cases of infection and therefore contact with children should not be discouraged.

The current study had several strengths. It was designed so that demographic and health data could be objectively verified with patient charts and through conversations with patients which increased reliability of the data collected. Surprisingly there was a very low cumulative discrepancy rate (1.7%) for variables that were cross-referenced between the survey and medical records. Since patients enrolled had to have MyChart, it is possible that patients viewed their medical record to retrieve medical information (e.g. medications, treatments, physicians notes) while answering questions on the survey. We did not track who did and did not do this; however, this may have made the medical-related answers on the survey reliable even prior to cross-referencing. In addition, collection of extensive demographic and health data allowed for the control of potential covariates and confounders in statistical analyses. Results in this study displayed the importance of controlling for these potential covariates and confounders and the avoidance of type I error, which may explain the inconsistent results with previous studies on this topic.

The current study also had limitations. As with all survey studies, self-report survey data can be bias. We were not able to objectively verify the data collected for the primary outcomes (i.e. time spent in contact with children and number of children); however, we were able to verify the majority of patient demographic and health data. Furthermore, the most severe outcome, death, could not be assessed as we did not survey family members of the deceased. In addition, we only surveyed positive patients and therefore could not assess whether having children protects against getting infection at all as Wood *et al*. [17] demonstrated. A general limitation of studying this topic in this population is that the demographics of persons most likely to have contact with children (i.e. aged <50 years) generally fall into established low-risk groups for COVID-19 [24], thus it is difficult to establish if being around children is independently associated with lower outcome severity. We were able to adjust for age as a covariate, minimizing inherent bias. Finally, of the 77 427 patients that tested positive for COVID-19 at Hartford HealthCare testing sites, 54% were female. In the current study, 72% of patients were female and therefore there was some gender ratio discrepancy between the enrolled study population and the larger population from which the study population derived from. However, we also adjusted for gender as a covariate, minimizing inherent bias.

Future research is needed examining patients with milder cases to explore other coronavirus immunity. In addition, more severe cases should be studied by surveying deceased patients’ family members in order to examine the association between contact with children and mortality due to COVID-19 [25]. Additional population surveys of seropositive but asymptomatic cases are needed to examine if contact with children is related to their benign course or perhaps protective against any infection at all, while adjusting for potential confounders and covariates. Finally looking closer at thromboembolism and child contact may be appropriate based on our limited but intriguing finding.

Although the advent and success of the COVID-19 vaccinations are protective against severe disease, there remains a possibility of new resistant strains. Therefore, the potential of any pre-protection conferred by child contact leading to the mitigation of disease severity will remain relevant and deserves further study.

## Data Availability

Data are available by request to the corresponding author (PJ) with permission from the IRB and Principal Investigators.

## Supplementary material

For supplementary material accompanying this paper visit https://doi.org/10.1017/S0950268822000474. click here to view supplementary material
