# Supplementary material for: The association between contact with children and the clinical course of COVID-19
Source: Epidemiol Infect. 2022 Mar 7;150:e64. doi: 10.1017/S0950268822000474 (PMC8943224; doi:10.1017/S0950268822000474)
Supplement: Supplementary file 1 [file S0950268822000474sup001.docx]

Thank you for agreeing to participate in this short survey. The survey will take approximately 10 minutes to complete and will ask you questions about your demographics, medical information related to COVID-19, and the amount of time you spend with children at work and at home. The final question will ask if you would like to be entered into a random drawing for a chance to be one of 20 participants that will be selected to win an iPad.

**A. Demographics**

1) What is your age?

2) What is your gender?

3) What is your approximate height? Feet _____ Inches______

4) What is your approximate weight in pounds?

**B. Medical Information Related to Covid-19 Diagnosis**

5) During what month did you test positive or were diagnosed with COVID-19?

6) What symptoms did you have related to your COVID-19 diagnosis? Select all that apply:

- Fever or chills
- Cough
- Shortness of breath or difficulty breathing
- Fatigue
- Muscle or body aches
- Headache
- New loss of taste or smell
- Sore throat
- Congestion or runny nose
- Nausea or vomiting
- Diarrhea

7) Were you hospitalized due to COVID-19? Select yes or No. If No, questions 8 through 10 are not applicable.

8) How many days were you hospitalized?

9) To the best of your knowledge, did your COVID-19 related complications include any of the following? Select all that apply:

- Pneumonia
- Respiratory failure
- Sepsis and septic shock
- Cardiomyopathy and arrhythmia
- Kidney injury
- Thromboembolism
- Gastrointestinal bleeding
- Critical illness polyneuropathy/myopathy
- Other. Please describe. __________________
- I did not have any complications
- I am not sure

10) Which best describes your treatment while at the hospital? Select all that apply.

- I was given medications only
- I was given medications and put on a ventilator
- My care took place in the intensive care unit
- My care took place on the floor unit (where you are cared for when you do not require very close monitoring)
- Other. Please describe. _____________________________

11) Prior to your COVID-19 diagnosis, did you exercise regularly?

Regular exercise is defined as participating in planned, structured physical activity for at least 30 minutes at moderate intensity on at least 3 days per week for at least the last 3 months. Select yes or no.

12) Following your COVID-19 diagnosis, did you exercise regularly? Select yes or no.

13) At the time of your COVID-19 diagnosis, did you have any of the following medical conditions? Select all that apply.
 High Blood Pressure
 Diabetes
 Asthma
 Congestive Heart Disease
 Any Autoimmune Disorder
 Any Chronic Lung Disease (COPD, emphysema, long term cigarette use)
 Chronic Renal Failure

14) Do you take any medications regularly (including over the counter medications and vitamins)? Select yes or no. If yes, please indicate what those medications are.______________

**C. Exposure to Children at Your Workplace**

15) Do you currently or have you had contact with children at your job since January, 2020?

Select yes or no. If no is selected, questions 16 through 19 are not applicable.

16) What is your job in which you work with or are in contact with children? Some examples include: teacher, daycare worker, children’s health care provider.

17) At your job, **how many hours per week** do you estimate your contact is with children?

18) At your job, approximately **how many children per day** are you in contact with?

19) At your job, what are the ages of the children you have contact with? Select all that apply.

 0-3
 4-6
 7-9
 10-14
 15-20

**D. Exposure to Children at Your Home**

20) Do you currently or have you had contact with children in your household since January, 2020? Select Yes or No. If No is selected, questions 21 through 25 are not applicable.

21) What best describes the nature of your contact with children at home?

- Work from home full time with children home
- Work from home part time with children home
- Full time stay at home parent or guardian
- Parent or guardian who cares for children during off-work hours (for example, evenings, nights, and weekends).
- Other. Please describe._____________________________

22) Would you consider yourself the primary care taker for the children you live with? Select yes or no.

23) At home, **how many hours per week** do you estimate your contact is with children?

24) At home, how many children do you live with?

25) At home, what are the ages of children you live with? Select all that apply.

 0-3
 4-6
 7-9
 10-14
 15-20

**E. Electronic Medical Record Permissions**

26) Would you like to give the study Investigators permissions to access your electronic medical record in order to confirm your answers to the medical related questions? Confirming the answers to survey questions using your medical record will help us to ensure the accuracy of the data we are collecting. Select Yes or No.

**F. Random Drawing**

27) Would you like to be entered into a random drawing for a chance to be one of 20 participants to win an iPad? Select Yes or No.
